# Supplementary material for: Reconciling Mining with the Conservation of Cave Biodiversity: A Quantitative Baseline to Help Establish Conservation Priorities
Source: PLoS One. 2016 Dec 20;11(12):e0168348. doi: 10.1371/journal.pone.0168348 (PMC5173368; doi:10.1371/journal.pone.0168348)
Supplement: S1 Dataset — (ZIP) [file pone.0168348.s002.zip › Taxa/Serra Norte/SN_2013/N4E/Lista N4E-51.pdf]

CAVIDADE N4E-0051

Classe	Ordem	Fam/Outros	Gên/Outros	Espécie	S-U	U-S	Zon.
Arachnida	Acari	Ascidae		sp.2		X	P
Arachnida	Acari	Ixodidae	*Ixodes*	sp.1	X		P
Arachnida	Acari	Mesostigmata		jovem	X		P
Arachnida	Acari	Prostigmata		jovem	X		P
Arachnida	Acari	Rhagidiidae		sp.20	X		P
Arachnida	Acari	Trombiculidae		sp.41	X		P
Arachnida	Amblypygi	Phrynidae	*Heterophrynus*	*longicornis*	1	2	E/P
Arachnida	Araneae	Araneidae		jovem		X	E
Arachnida	Araneae	Caponiidae		jovem		X	E
Arachnida	Araneae	Filistatidae		jovem	X		E
Arachnida	Araneae	Ochyroceratidae	*Ochyrocera*	sp.1	X		P
Arachnida	Araneae	Ochyroceratidae	*Ochyrocera*	sp.3		X	P
Arachnida	Araneae	Ochyroceratidae	*Speocera*	sp.1	X		P
Arachnida	Araneae	Oonopidae	aff. *Xyccarph*	sp.2		X	E
Arachnida	Araneae	Pholcidae	Ninetinae	sp.1	X	X	E/P
Arachnida	Araneae	Salticidae		sp.2	X	X	E
Arachnida	Araneae	Segestriidae		jovem		X	E
Arachnida	Araneae	Tetragnathidae		jovem		X	E
Arachnida	Araneae	Theridiidae	*Theridion*	sp.1		X	E
Arachnida	Araneae	Theridiosomatidae	*Plato*	sp.1	X	X	P
Arachnida	Opiliones	Cosmetidae	*Roquettea*	*singularis*	1		
Arachnida	Opiliones	Stygnidae	*Protimesius*	*gracilis*	2		
Arachnida	Pseudoscorpiones	Chernetidae	*Spelaeochernes*	sp.1	X		P
Arachnida	Scorpiones	Buthidae	*Ananteris*	sp.1	1		
Chilopoda	Geophilomorpha	Mecistocephalidae	*Mecistocephalus*	sp.1		1	E
Diplopoda	Polydesmida	Pyrgodesmidae		sp.1		X	P
Entognatha	Collembola	Paronellidae		sp.1	X	X	P
Entognatha	Collembola	Paronellidae		sp.4	X		P
Insecta	Blattodea	Polyphagidae		jovem		1	E
Insecta	Diptera	Cecidomyiidae	Cecidomyiinae	sp.		X	E
Insecta	Diptera	Chironomidae		sp.	X		P
Insecta	Diptera	Dolichopodidae		sp.	X		P
Insecta	Diptera	Drosophilidae	*Drosophila*	*eleonorae*	X		E
Insecta	Diptera	Psychodidae	Phlebotominae	sp.		X	E/P
Insecta	Diptera	Tipulidae	Tipulinae	sp.	X	X	E/P
Insecta	Heteroptera	Cydnidae		sp.		X	P
Insecta	Heteroptera	Dipsocoroidea		sp.	X		P
Insecta	Heteroptera	Lygaeidae		jovem		X	E
Insecta	Heteroptera	Nabidae		sp.		X	E
Insecta	Homoptera	Cixiidae		sp.3	X		E
Insecta	Hymenoptera	Formicidae	*Acromyrmex*	sp.1	X		P
Insecta	Hymenoptera	Formicidae	*Dolichoderus*	aff. *bispinosus*	X		E/P
Insecta	Hymenoptera	Formicidae	*Gnamptogenys*	sp.1	X		P
Insecta	Hymenoptera	Formicidae	Myrmicinae	sp.1		X	P
Insecta	Hymenoptera	Formicidae	*Odontomachus*	sp.3		X	P
Insecta	Hymenoptera	Formicidae	*Pachycondyla*	sp.2	X		E
Insecta	Hymenoptera	Formicidae	*Pheidole*	sp.1		X	P
Insecta	Hymenoptera	Formicidae	*Pheidole*	sp.3		X	E
Insecta	Hymenoptera	Formicidae	*Trachymyrmex*	sp.1	X		P

Insecta	Isoptera	Termitidae	*Atlantitermes*	sp.		X	E
Insecta	Lepidoptera	Roeslerstammiidae		sp.		X	E
Insecta	Orthoptera	Gryllidae	Nemobiinae	jovem		1	P
Insecta	Orthoptera	Phalangopsidae	*Paraclodes*	sp.	3	3	E/P
Insecta	Orthoptera	Phalangopsidae	*Phalangopsis*	sp.	4	12	P
Insecta	Psocoptera	Epipsocidae		jovem	X		P
Gastropoda	Pulmonata	Bulimulidae	*Naesiotus*	sp.	X		P
Malacostraca	Isopoda	Dubioniscidae		sp.1		X	E
Malacostraca	Isopoda	Scleropactidae	*Circoniscus*	*buckupi*		X	P
Amphibia	Anura	Strabomantidae	*Pristimantis*	cf. *fenestratus*		2	P
Mammalia	Chiroptera	Emballonuridae	*Peropteryx*	*kappleri*	1		E
